# IPO11 regulates the nuclear import of BZW1/2 and is necessary for AML cells and stem cells

**DOI:** 10.1038/s41375-022-01513-4

**Published:** 2022-02-12

**Authors:** Boaz Nachmias, Dilshad H. Khan, Veronique Voisin, Arvind S. Mer, Geethu Emily Thomas, Nadav Segev, Jonathan St-Germain, Rose Hurren, Marcela Gronda, Aaron Botham, Xiaoming Wang, Neil Maclean, Ayesh K. Seneviratne, Nathan Duong, Changjiang Xu, Andrea Arruda, Elias Orouji, Arash Algouneh, Razqallah Hakem, Liran Shlush, Mark D. Minden, Brian Raught, Gary D. Bader, Aaron D. Schimmer

**Affiliations:** 1 Princess Margaret Cancer Centre, University Health Network, Toronto, ON Canada; 2 Department of Hematology, Hadassah Medical Center and Faculty of Medicine, Hebrew University of Jerusalem, Jerusalem, Israel; 3 Terrence Donnelly Centre for Cellular and Biomedical Research, University of Toronto, Toronto, ON Canada; 4 Dept. of Biochemistry, Microbiology and Immunology, Faculty of Medicine, University of Ottawa, Ottawa, ON Canada; 5 Department of Medical Biophysics, University of Toronto, Toronto, ON Canada; 6 Department of Immunology, The Weizmann Institute of Science, Rehovot, Israel

**Keywords:** Leukaemia, Cell signalling

## Abstract

AML cells are arranged in a hierarchy with stem/progenitor cells giving rise to more differentiated bulk cells. Despite the importance of stem/progenitors in the pathogenesis of AML, the determinants of the AML stem/progenitor state are not fully understood. Through a comparison of genes that are significant for growth and viability of AML cells by way of a CRISPR screen, with genes that are differentially expressed in leukemia stem cells (LSC), we identified importin 11 (IPO11) as a novel target in AML. Importin 11 (IPO11) is a member of the importin β family of proteins that mediate transport of proteins across the nuclear membrane. In AML, knockdown of IPO11 decreased growth, reduced engraftment potential of LSC, and induced differentiation. Mechanistically, we identified the transcription factors BZW1 and BZW2 as novel cargo of IPO11. We further show that BZW1/2 mediate a transcriptional signature that promotes stemness and survival of LSC. Thus, we demonstrate for the first time how specific cytoplasmic-nuclear regulation supports stem-like transcriptional signature in relapsed AML.

## Introduction

Acute myeloid leukemia (AML) is a heterogeneous clonal disorder characterized by the dysregulated proliferation and differentiation arrest, resulting in the accumulation of immature myeloid progenitors in the bone marrow and peripheral blood. While most patients achieve remission with initial therapy, the majority relapse leading to poor overall survival [1]. Relapse is frequently driven by a rare subset of leukemic stem cells (LSC) [2, 3]. LSC maintain stem-like transcriptional signatures with self-renewal ability, specific metabolic features [4, 5] and resistance to chemotherapy [6]. Therefore, understanding the biological mechanisms that maintain LSC should help to identify new therapeutic strategies for this disease. Herein we report the identification of IPO11, a regulator of protein trafficking between the nucleus and cytoplasm, as a mediator of stemness in LSC.

Proteins transit into and out of the nucleus through the nuclear pore complex (NPC). The NPC is composed of multiple copies of up to 50 different proteins termed nucleoporins [7]. The bidirectional transport of proteins across the NPC is mediated by nuclear transport receptors that recognize cargo-protein either by their nuclear localization signal/sequence or nuclear export signals. Nuclear transport receptors have protein domains that bind to the import or export localization signal of their cargo, the NPC, and the small Ras-like GTPase Ran, that maintains the driving polarity across the nuclear membrane [8, 9]. Some nuclear transport receptors directly bind their cargo while others require an adapter protein [10]. Nuclear transport receptors are divided into importins and exportins depending on the direction of transport.

The most extensively studied exportin is Exportin 1/chromosome region maintenance 1 (XPO1/CRM1). XPO1 mediates nuclear export of a wide variety of proteins, including p53, p27, NPM, FOXO1, IkB, Rb, PI3K, and AKT [11]. XPO1 is upregulated in many cancer types and its inhibition disrupts many of key processes in tumorigenesis [12]. The first-in-class XPO1 inhibitor selinexor, has shown anti-proliferative effect both in vivo and in vitro in AML and myeloma, and is approved for the treatment of myeloma [13–15].

While the export of nuclear proteins has been studied extensively, less is known about the import of nuclear proteins. To date, 10 importin family members have been identified and each carrier imports specific protein cargo. However, the biological roles of individual importins and their necessity for the survival of malignant cells and stem cells are not understood. Here, we investigate importin 11 (IPO11) as an important regulator of LSC function and survival.

## Materials and methods

### Bioinformatic analysis

*Haferlach AML cohort*. Affymetrix gene expression data of AML (542 samples) and healthy bone marrow samples (73 samples) from the Haferlach data set (GSE13159) was downloaded from the leukemia-gene atlas (http://www.leukemia-gene-atlas.de) [16]. *Diagnosis/Relapse AML cohort*. Gene expression of diagnosis and relapse AML samples were obtained from a previous study by Shlush et al. [2]. *TCGA cohort*. Raw counts from 179 AML samples from the LAML TCGA data were downloaded from the GDC portal (https://portal.gdc.cancer.gov/). AMLs were categorized into Roc/progenitor-like or Rop/myeloid-like as described [2]. *beatAML cohort*. CPM normalized counts of 451 AML samples were obtained from Tyner et al. [17]. *LSC+/LSC− and CD34+CD34− AML gene expression data*. Illumina beadchip transcriptomics data containing LSC+, LSC−, CD34+ and CD34− sorted AML fractions were obtained from the Gene Expression Omnibus data portal (GSE76008) [18]*. Ramalho data set*. MsigDb gene set contains 197 up and 74 downregulated genes defining stem cells [19]. Details of data analysis are described in supplementary methods.

### Cell lines

OCI-AML2 cells were grown in Iscove’s Modified Dulbecco’s Medium (IMDM). NB4 cells were grown in RPMI media. Both media were supplemented with 10% fetal bovine serum and appropriate antibiotics. TEX leukemia cells and 8227 cells were obtained from Dr. John Dick’s lab [20, 21]. TEX cells were maintained in IMDM with 20% FBS, 2 mM l-glutamine, 20 ng/mL stem cell factor (SCF), and 2 ng/mL interleukin-3 (IL-3); 8227 and primary cells were grown in X-VIVO-10 with 20% bovine serum albumin-insulin-transferrin (BIT), Fms-related tyrosine kinase 3 ligand (Flt3-L, 50 ng/ml), interleukin-6 (IL-6, 10 ng/ml), SCF (50 ng/ml), thrombopoietin (TPO, 25 ng/ml), IL-3 (10 ng/ml), granulocyte colony-stimulating factor (G-CSF, 10 ng/ml). All cell lines were maintained at 37 °C, supplemented with 5% CO_2_. Cell lines were periodically tested for mycoplasma. Information about the patients who were the source of the cells is indicated in Table S3. The University Health Network institutional review boards approved the collection and use of human tissue for this study (Research Ethics Board protocol #13-7163).

### CRISPR knockout

The sequence for IPO11 (gene id: 51194) gRNA1: 5′-CAAATCACCCTGCGTCGCAA-3′; IPO11-gRNA2: 5′- ATGACGGAAGATCCTGAAAC-3′; Control-gRNA, LacZ:5′-CCCGAATCTCATCGTGCGG-3′ were cloned into pLCKO lentiviral vector, as described previously [22].

### Plasmids and cell transductions

#### shRNA knockdown

shRNA constructs in the hairpin-pLKO.1-Puro vector were purchased from Sigma-Aldrich^®^ as glycerol bacterial stocks. To transduce shBZW2 hairpin-pLKO.1 MISSION^®^ custom vector with G418 selection was used. For 8227 cells and primary AML patient samples, IPO11 shRNA sequences were first modified in order to be cloned into the hairpin-pRS19 vector using the restriction enzyme BsbI.

pLENTI-C-HA-DDK-P2A-PURO HA-tagged BZW1 (NM_001207067), BZW2 (NM_001159767), pLenti-EF1a-C-myc-DDK_IRES IPO11 (NM_016338), and TYK2 (NM_003331) were purchased from Blue Heron.

The shRNAs sequences, lentiviral packaging, and infections are described in Supplementary methods.

### Cell growth and colony-forming assays

Cell count was by the automated cell counter ADAM (Montreal Biotech), according to manufacturer protocol. For the TYK2 inhibitor experiments, cell proliferation was performed using as XTT-based kit (Cell proliferation kit, Biological Industries, Israel). Cells were plated in a flat-bottom 96-well plate at 10,000 cells per well and treated with the TYK2 inhibitor (BMS-986165; MCE, USA) for 72 h. Colony-forming assays were done as described before [5]

### Cell lysates preparation and western blot analysis

Whole-cell protein lysates were obtained with RIPA buffer. The Nuclei EZ prep nuclei isolation kit was used for cytoplasmic and nuclear fractionation, (Sigma-Aldrich, Missouri, USA), as per the manufacturer’s instructions. Primary antibodies included anti-IPO11 (OriGene AP), anti-BZW1 (Proteintech 19917-1-AP), anti- p21 (Cell Signaling #2947), anti-actin (Santa Cruz sc-69879), anti-Histone 3 (Cell Signaling #4499), anti-TYK2 (Cell Signaling #14193), anti-phospho-γH2Ax (EMD Millipore, 05-636).

### Flow cytometry

NB4 cells were stained with anti-human CD11b APC (BD340937) and 7AAD (BD559925). 8227 cells were co-immunostained with Annexin V (BD Biosciences, BD 556419), and anti-human antibodies recognizing CD34 (BD Biosciences, BD 340411) and CD38 (ThermoFisher Scientific 12-0388-42). Flow cytometry data were acquired using a BD Accuri flow cytometer (BD Biosciences, FL, USA) and frequency of viable 34+, 38− cells analyzed with the FlowJo software (TreeStar, OR, USA).

### Animals

Twelve-week male or female immunodeficient NOD.Cg-Prkdc^scid^ Il2rg^tm1Wjl^ Tg (CMV-IL3, CSF2, KITLG)1Eav/MloySzJ (NOD-SCID-GF) mice were obtained from Dr. Connie J. Eaves and bred in our facility [23]. Twelve-week female immunodeficient NOD.CB17-Prkdc^scid^/J (NOD-SCID) were obtained from the University Health Network.

For the in vivo experiments with mice, the mice were grouped prior to treatment. The grouping and treatment of the mice were performed by an individual who was not involved in the analysis of the data from the experiment. Mice were randomly assigned to each experimental group. During all experiments, the weights of the mice were approximately 18–30 g with no animals losing greater than 10% body weight. All animals were housed in microisolator cages with temperature-controlled conditions under a 12-h light/dark cycle with free access to drinking water, and food. Data were not excluded.

All animal studies were performed in accordance with the University Health Network Animal Use Protocol: #1251.34.

### TEX, 8227, and primary AML cells engraftment

Equal numbers of TEX and 8227 cells (2 × 10^5^) were transduced with the specified IPO11 shRNA sequence or non-target control sequences and injected into the right femur of sub-lethally irradiated NOD-SCID-GF [5, 23]. The mice were also injected with 200 μg per mouse of anti-mouse CD122 antibody. To measure leukemic engraftment, five weeks after injection, mice were sacrificed, cells were flushed from the left femur, and the percentage of human CD45+ determined by flow cytometry. To measure survival, mice were monitored for signs of morbidity.

For primary AML cells, 1 × 10^6^ cells were transduced with shIPO11 or non-target control and injected into the right femur of NOD-SCID mice. GFP positive rate was determined by flow prior to injection. Eight weeks after, mice were sacrificed, cells were flushed from the left femur and the CD33+/CD45+ GFP+ population was determined by flow cytometry. Relative engraftment was measured as previously described, to account for differences in the initial GFP+ percentage in the control versus knockdown samples [5]. For all of the experiments performed the GFP+ difference was below 5%. For secondary engraftment, equal numbers of cells derived from the left femur of the primary engraftment experiment were reinjected into the right femur of untreated mice. Eight weeks later, mice were sacrificed, cells were flushed from the left femur and the CD33+/CD45+ GFP+ population was determined by flow cytometry.

### Non-specific Esterase stain

NSE stain kit from Sigma-Aldrich was used, was used as per manufacturer’s instructions. Slides were scanned by the Aperio ScanScope AT2 (Leica, Wetzlar, Germany). Five random sections were analyzed by ImageJ for NSE intensity.

### qRT-PCR

Details on Quantitative real time polymerase chain (qRT-PCR) reactions are described in supplementary methods.

### RNA sequencing and analysis

RNA was isolated from OCI-AML2 cells 7 days post-transduction with shRNA targeting IPO11, dual BZW1/2 compared and control and were sequenced (Illumina Nextseq2500). Details of data analysis are described in Supplementary methods. Raw data was deposited in GEO repository, GSE173288.

### ATAC sequencing and analysis

Control or IPO11 knockdown OCI-AML2 cells were prepared as described above. Purified libraries were evaluated for enrichment by qPCR using primers designed against open regions (KAT6B and GAPDH) compared against closed regions (QML93 and SLC22A3). Details of data analysis are described in supplementary methods.

### BioID assay-proximity-dependent biotinylation

BioID and mass spectrometry were conducted as described previously [24].

### Confocal microscopy

Cyto-spin slides were prepared by centrifuging 1 × 10^5^ cells for 5′ at 1000 rpm. Cells were then fixed with 2% paraformaldehyde for 5′ at room temperature (RT). Next, cells were incubated in blocking and permeabilization solution of PBS 5% BSA, 0.1% Triton-X, for 30′ in RT. BZW1 antibody (Proteintech) 1:200 in the above solution was added to the fixed cells for 1 h at RT followed by three washes (1.5 ml of the above solution). Anti-rabbit Alexa-488 conjugated antibody (Jackson Immuno-research) was added for 1 hour, followed by three washes. DAPI (1:1000) was added for 1′ at RT in PBS. Finally, glass slides were mounted with anti-fade reagent, dried at RT overnight and analyzed using a Leica SP8 confocal microscope.

### ChIP sequencing

ChIP assays were performed using OCI-AML2 cells expressing either HA-tagged BZW1 or BZW1 as previously described [25], with a minor modification, in that cross-liked chromatin was sheared to ~500 bp fragments by sonication. Antibody against anti-HA (Abcam) and BZW1 (info) were used. ChIP DNA was sequenced on Illumina Nextseq2500. Raw data were deposited in GEO repository, GSE173288.

### Statistical analysis

GraphPad Prism 6.0 was used to perform analyses. Statistical analyses were performed by unpaired student’s *t*-test or one-way ANOVA test. Statistical significance was defined as *p* < 0.05. Variability was comparable between groups in animal and in vitro studies. Sample sizes were chosen based on the feasibility of the number of replicates that could be managed in an experiment and past experiences.

## Results

### IPO11 is a critical target in leukemic stem cells

**Fig. 1:**
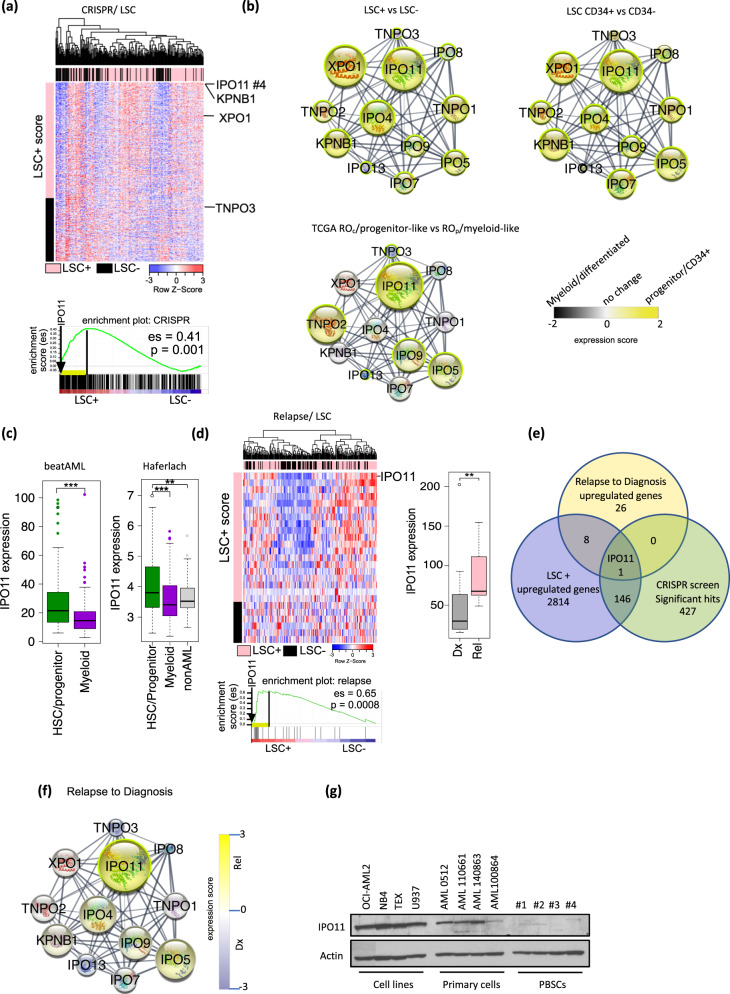
Identification of IPO11 as a novel target in AML. **a** A genome-wide CRISPR of 91,320 barcoded gRNA in Cas9-OCI-AML2 cells identified depleted sgRNAs that are essential for AML. Positive-hits were identified at a false discovery rate (FDR) < 5%. Genes were ranked based on MAGeCK scores [Log2 of p-values] as calculated by the MAGeCK algorithm. Significant genes in the CRISPR screen were correlated with genes upregulated in AML engrafted LSC fractions. GSEA plot shows significant enrichment of essential genes from CRISPR screen in LSC fractions. **b** Cytoscape STRING interaction network of IPO11. The lines (edge) between the circles (nodes) represent know functional interactions. The yellow color versus black represents a high expression of IPO11 in AML LSC+ vs. LSC− (upper left panel, FDR ≤0.01, GSE76008), AML CD34+ vs. CD34- (upper right panel, FDR ≤ 0.01, GSE76008), progenitor/stem vs myeloid cluster (lower left, FDR ≤ 0.01, TCGA-AML). Node (circle) size is proportional to expression. Significance (FDR ≤ 0.01) of differential gene expression are highlighted by a green node border. **c** IPO11 expression in AML classified as myeloid and HSC/progenitor as well as normal hematopoietic cells in the specified database. AML classification method is further specified in supplementary Fig. S1c. **d** Upregulated genes at relapse compared to diagnosis in 11 paired samples (FDR < 0.05%) were correlated with gene upregulated in AML engrafted LSC fractions. GSEA plot shows enrichment of genes upregulated in relapse in LSC. Adjacent box-plot demonstrates IPO11 is significantly upregulated in relapse samples (FDR <= 0.01). **e** Venn diagram overlaying essential genes from CRISPR (FDR <= 0.01) with genes that are upregulated in LSC and relapse at FDR 0.01. **f** Cytoscape STRING interaction network of importin β family members in paired diagnosis/relapse samples. Increasing yellow color represents higher expression of IPO11 in relapse vs. diagnosis. Significance (FDR ≤ 0.01) of differential gene expression are highlighted by a green node border. **g** Expression of IPO11 protein in cell lysates form AML cell lines, primary AML cells, and normal hematopoietic cells.

To identify genes that are essential for the viability of LSC, we overlaid the results of our previously performed, genome-wide CRISPR screen [5] with the expression of genes enriched in functionally defined LSCs [18]. Through that CRISPR screen, we identified 570 genes whose knockout reduced the growth and viability of OCI-AML2 cells (FDR < 0.01). By overlaying the hits from our CRISPR screen with LSC enriched gene expression, we identified the importin β family member, IPO11, as a top hit, with a 7.5-fold increase in RNA expression in the LSC+ fraction as compared to LSC- fractions (Fig. 1a). IPO11 is a member of the importin β family of proteins that mediates cargo shuttling across the nuclear membrane, and bears an N-terminal Ran-binding domain similar to other family members [26, 27]. Only three hits from our CRISPR screen were expressed at higher levels in the LSC+ fraction compared to IPO11: *RPAP2*, *KDSR*, and *CDK6*. *RPAP2* and *KDSR* have previously been linked to stem cell function and differentiation [28, 29] and *CDK6* is a known regulator of LSC function and clonogenicity [30] (Supplemental Table S1). As to date, IPO11 has not recognized as necessary for the viability of AML cells, we focused our study on IPO11.

The level of IPO11 in publicly available data sets showed that the level of expression was higher in LSC+ (engrafting) versus LSC- (non-engrafting) primary AML samples (FDR ≤ 0.05, GSE76008). IPO11 expression was also higher in CD34+ vs CD34- AML fractions (FDR ≤ 0.05, GSE76008, Fig. 1b and Supplemental Fig. S1a, b). IPO11 was increased in undifferentiated progenitor vs. more differentiated myeloid clusters from three additional AML datasets. Finally, expression of IPO11 was higher in AML compared to normal hematopoietic cells (FDR <= 0.05, Fig. 1b, c and Supplemental Fig. S1c, d). In two public datasets of gene expression and AML patient outcome (TCGA and Beat AML), increased expression of IPO11 was associated with a significant, but modest lower overall survival (Supplemental Fig. S2a). High levels of IPO11 also correlated with poor ELN risk [31] and a high LSC-17 score [18], both known negative predictive factors in AML (Supplemental Fig. S2b). High expression of IPO11 correlated with mutations in *c-KIT*, *DNMT3A* and *NRAS* (*p* value < 6E–9, Supplemental Table S2).

RNAseq was carried out for 11 pairs of AML samples obtained at diagnosis and relapse, with clear enrichment of LSC+ genes in relapse [2]. IPO11 was one of 34 genes increased at relapse in all paired samples (Fig. 1d and Supplemental Fig. S2c) and the only one of the 34 genes that was enriched in the LSC+ fraction and necessary for viability as determined in our CRISPR screen (Fig. 1e).

To date, among the family of importin and exportin nuclear shuttling proteins, only exportin 1 (XPO1) has been studied in AML, where it is important for AML and LSC survival [32]. Indeed, XPO1 was a hit in our CRISPR screen, and its expression was increased in LSC versus bulk cells, albeit not to the extent of IPO11 (Fig. 1b and Supplemental Fig. 2d). The canonical importin protein, importin β1, KPNB1, also ranked high on our CRISPR screen, but had a much lower enrichment in the LSC (3.6-fold) compared to bulk leukemia fractions (Fig. 1b). Notably, neither XPO1 or importin β1 were upregulated at relapse in the paired diagnosis/relapsed samples (Fig. 1f and Supplemental Fig. S2e).

Finally, we confirmed increased expression of IPO11 protein by immunoblotting in AML cell lines and primary AML cells compared to normal hematopoietic cells (Fig. 1g). Please see Supplementary Table S3 for patients’ clinical data.

#### IPO11 is necessary for AML growth and viability

**Fig. 2:**
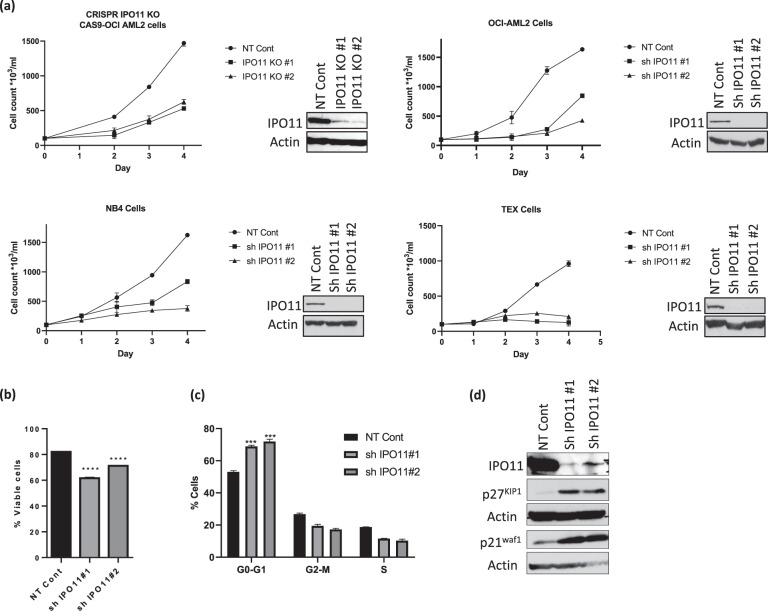
IPO11 is necessary for the growth and viability of AML cells. **a** Cas9 expressing OCI-AML2 were transduced with gRNA targeting IPO11. Seven days after transduction, cell viability and proliferation were measured with an automated fluorescent cell counter. IPO11 and actin expression were measured by immunoblotting. OCI-AML2, NB4, and TEX cells transduced with shRNA targeting IPO11. Four days after transduction of shRNA, cell viability and proliferation were measured with an automated fluorescent cell counter. IPO11 and actin expression were measured by immunoblotting. **b** Cell viability in OCI-AML2 cells 4 days after IPO11 knockdown was measured by Annexin V/PI staining. ****p ≤ 0.0001, by *t*-test. **c** Cell cycle analysis in OCI-AML2 cells 4 days after IPO11 knockdown was measured by PI staining. ***p ≤ 0.001, by *t*-test. **d** Expression of p27^kip1^ and p21^waf1^ in OCI-AML2 cells 4 days after IPO11 knockdown.

To study the functional importance of IPO11 in AML, we knocked out IPO11 with CRISPR and individual gRNAs. IPO11 knockout reduced the growth and viability of OCI-AML2 cells by 80% (Fig. 2a, left upper panel). We also confirmed that partial target knockdown of IPO11 with shRNA reduced AML growth and viability of OCI-AML2, NB4 and TEX leukemia cells >80% (Fig. 2a). IPO11 knockdown decreased AML viability as measured by Annexin V/PI staining (Fig. 2b) and arrested cells at the G0/G1 transition (Fig. 2c). Consistent with the induced cell cycle arrest, the levels of cell cycle arrest markers p21^waf1^ and p27^kip1^ were increased upon IPO11 knockdown (Fig. 2d).

Knockdown of IPO11 did not change DNA damage induced by either radiation or daunorubicin (Supplementary Fig. S3a, b).

#### IPO11 knockdown impairs AML stem/progenitor function and increases differentiation

**Fig. 3:**
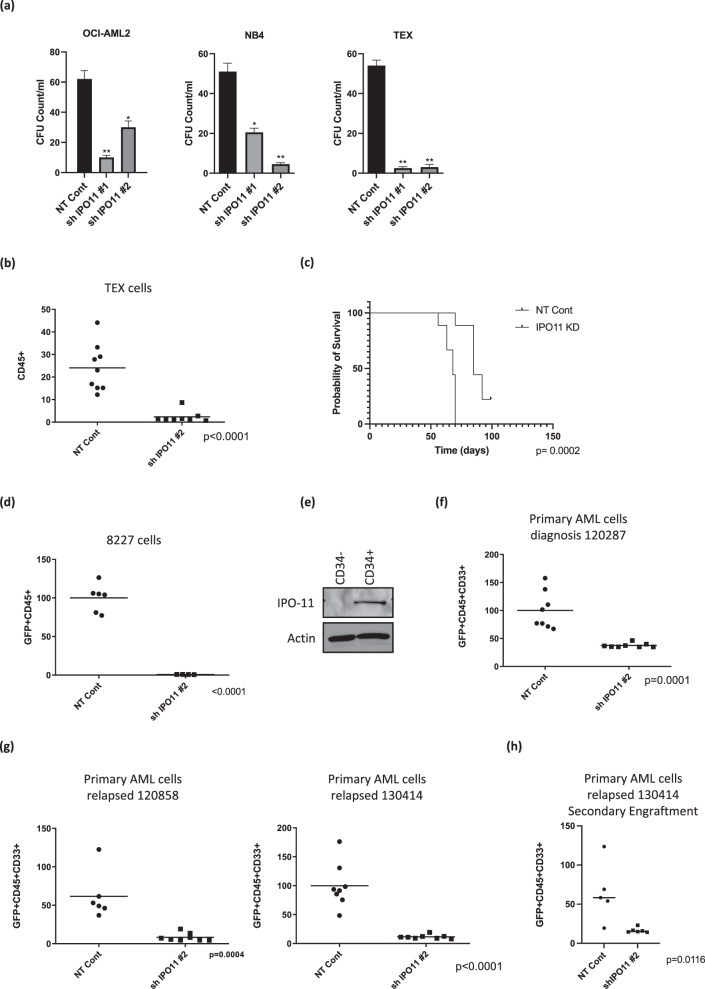
IPO11 is necessary for primary AML stem/progenitor cells. **a** Clonogenic growth of OCI-AML2, NB4 and TEX cells transduced with shRNA targeting IPO11 or control sequence. Mean ± SD colony counts are shown per ml (750 cells). *p ≤ 0.05, **p ≤ 0.01, by *t*-test. **b** TEX AML cells were transduced with shRNA targeting IPO11 or control sequences. 6 days after transduction, equal numbers of viable cells were injected into sub-lethally irradiated NOD/SCID-GF mice. The percentage of human CD45+cells in the non-injected femur was determined by flow cytometry (*n* = 8–9/group) after five weeks. The bar represents mean engraftment. *p* < 0.0001 by *t*-test. **c** TEX cells transduced with either non-target control or shRNA targeting IPO11 were injected into NOD.SCID-GF mice. Survival was measured over time. **d** 8227 cells were transduced with shRNA targeting IPO11 or control sequences in lentiviral vectors containing a GFP marker. Two days after transduction, equal numbers of viable cells were injected into the sub-lethally irradiated NOD/SCID-GF mice (*n* = 6 mice/group). Eight weeks later, the percentage of human GFP+, CD45+ cells in the non-injected femur was determined by flow cytometry. The transduction efficiency of the control and IPO11 shRNA lentiviral vectors were 30% and 32%, respectively. The bar represents mean engraftment, *p* < 0.0001 by *t*-test. **e** IPO11 expression in 8227 leukemia cells FACS sorted into bulk (CD34−) and stem cell (CD34+) fractions. **f** Primary AML cells were transduced with shRNA targeting IPO11 or control sequences in lentiviral vectors containing a GFP marker. Two days after transduction, equal numbers of viable cells were injected into sub-lethally irradiated NOD-SCID mice preconditioned with anti-CD122. Eight weeks after injection, the percentage of human GFP+, CD33+ and CD45+ cells in the non-injected left femur was determined by flow. **g** Engraftment of primary AML cells from relapse following IPO11 KD, same as described above. Transduction efficiency of NT control and IPO11 shRNA were 36% and 38% for patient 120878, 24% and 30% for patient 120858, 20% and 25% for patient 130414, respectively. IPO11 KD was confirmed by qPCR in GFP+ sorted cells. The bar represents mean engraftment. **h** Secondary engraftment of primary AML (130414) cells. Equal number of cells from mice in (**g**) were injected into the right femur of secondary untreated mice. Eight weeks after injection, the percentage of human GFP+, CD33+ and CD45+ cells in the non-injected left femur were determined by flow.

We next evaluated the effect of IPO11 knockdown on AML progenitors and stem cells. In OCI-AML2, NB4, and TEX leukemic cells, knockdown of IPO11 reduced clonogenic growth, consistent with an effect on leukemia initiating cells (Fig. 3a). TEX cells have properties of AML stem cells, including hierarchical structure and the ability to engraft into mouse marrow. We demonstrated that IPO11 knockdown decreased the engraftment of TEX cells in mouse marrow and prolonged their survival compared to mice injected with wild-type TEX cells (Fig. 3b, c). 8227 cells are a low passage primary AML culture model that are arranged in a hierarchy with the stem cells located in the CD34+CD38− fraction. Knockdown of IPO11 reduced the engraftment of 8227 cells into mouse marrow >90%, further demonstrating the necessity of this protein for AML stem/progenitors. (Fig. 3d). IPO11 was detected at the protein level only in the C34+ stem cell fraction, and not in the CD34− bulk leukemia fraction of 8227 cells (Fig. 3e).

Finally, we knocked down IPO11 in primary AML cells. IPO11 knockdown reduced engraftment of primary AML cells, including samples from patients with relapsed disease (Fig. 3f, g). IPO11 knockdown also reduced engraftment of primary AML cells in secondary transplants, further demonstrating the importance of IPO11 for the viability of AML stem cells (Fig. 3h, Supplementary Fig. S4) Thus, inhibiting IPO11 targets AML progenitors and stem cells, including those from relapsed patients.

**Fig. 4:**
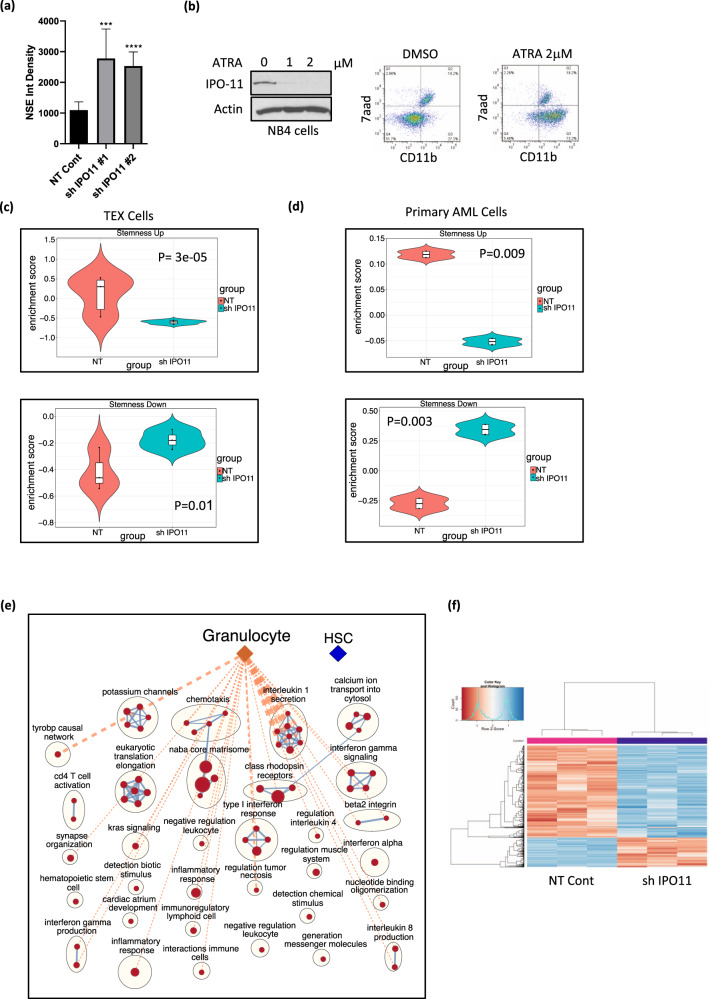
Silencing IPO11 promotes differentiation of AML cells. **a** Non-specific Esterase (NSE) staining in OCI-AML2 cells 7 days after transduction with shRNA targeting IPO11. The average of the 5 sections for each condition, from three independent experiments is presented. Data are mean ± SD. ***p ≤ 0.001 ****p ≤ 0.0001. **b** IPO11 protein expression after treatment of NB4 cells with increasing concentration of ATRA for 4 hours. Expression of CD11b by flow cytometry 4 h after treatment with 2 μM of ATRA. **c** Violin plots for changes in genes associated with AML stemness [19] in TEX cells. **d** Violin plots for changes in genes associated with AML stemness [19] in primary AML cells (120287). **e** Pathway analysis of differentially expressed genes IPO11 knockdown in OCI-AML2 cells. Red nodes: pathways significantly enriched in genes upregulated in IPO11 knockdown at FDR 0.05. post-analysis. **f** ATAC sequencing analysis of global accessible region number in NT cont vs. IPO11 KD in OCI-AML2 cells at FDR 0.05.

To test whether IPO11 knockdown affected AML differentiation, we measured changes in non-specific esterase (NSE). NSE staining was increased upon IPO11 knockdown (Fig. 4a). Moreover, IPO11 expression was reduced in NB4 cells after inducing their differentiation with all-trans-retinoic acid (ATRA) (Fig. 4b). In contrast, OCI-AML2 cells which do not differentiate after ATRA treatment, levels of IPO11 did not change after ATRA treatment (Supplementary Fig. S5a).

We next analyzed the change in gene expression and chromatin accessibility after IPO11 knockdown to further delineate the effect of IPO11 on stemness and differentiation. In AML cell lines knockdown of IPO11 shifted the gene expression profile away from LSC signatures and toward signatures associated with differentiated cells and granulocytes (Supplementary Fig. S5b). Similarly, in both TEX and primary AML cells, knockdown of IPO11 shifted gene expression away from a LSC+ signature [19] (Fig. 4c, d). By pathway analysis, knockdown of IPO11 increased genes associated with inflammation and immune response, which are also upregulated in differentiating myeloid cells (Fig. 4e). Finally, by ATAC sequencing, knockdown of IPO11 reduced regions of chromatin accessibility, a change associated with decreased stem cell properties and increased differentiation [33] (Fig. 4f).

#### BZW1 and BZW2 are IPO11 cargo

**Fig. 5:**
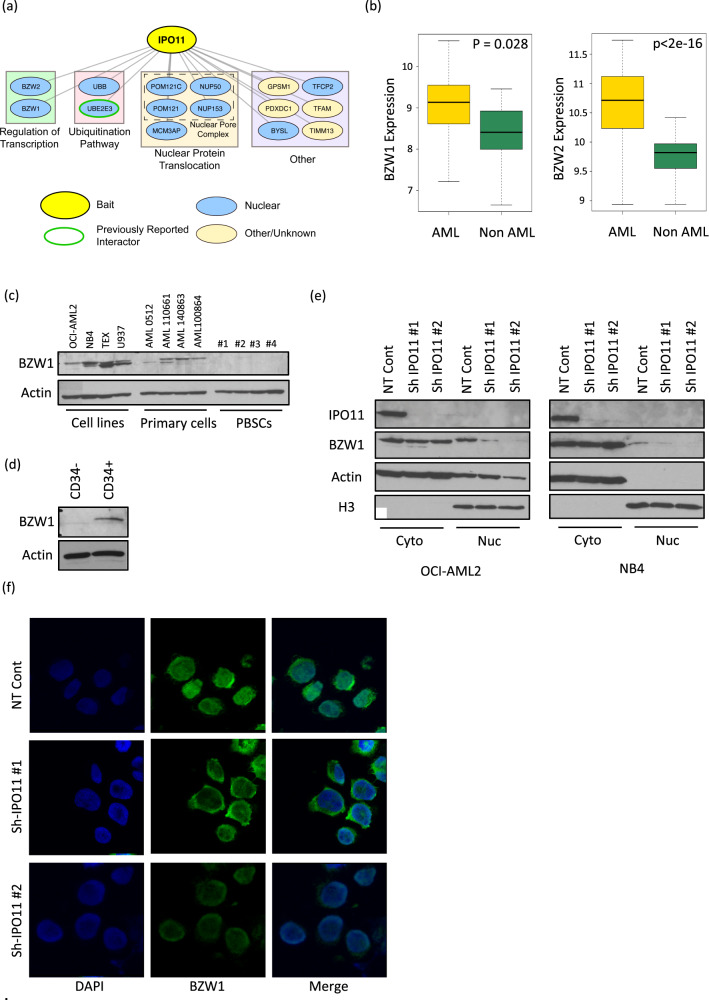
BZW1 and BZW2 are cargo of IPO11. **a** BioID identifies interactors with IPO11 (BFDR < 0.2). Pathways were manually annotated. **b** Expression of BZW1 and BZW2 in AML versus non-AML (Haferlach AML cohort). **c** BZW1 protein expression in cell lysates from AML cell lines, primary AML cells and normal hematopoietic cells. Actin loading control is the same as in Fig. 1g. **d** BZW1 protein expression in 8227 leukemia cells FAC sorted in to bulk (CD34−) and stem cell (CD34+) fractions. Actin loading control is the same as in Fig. 3e. **e** Levels of BZW1, IPO11, H3, and Actin protein in cytoplasmic and nuclear fractions isolated from OCI-AML2 and NB4 cells 4 days after transduction with shRNA targeting IPO11. **f** Expression of BZW1 by confocal microscopy in NB4 cells transduced with shRNA targeting IPO11. Blue- DAPI stain for nuclear visualization. Green- anti-BZW1.

To understand how IPO11 regulates AML stem cell function and differentiation and to better define its role of importing proteins into the nucleus, we sought to identify the cargo of IPO11 using proximity-dependent biotin labeling (BioID) coupled with mass spectrometry. We induced the expression of Flag-BirA*-IPO11 in HEK293T-REx cells and identified biotinylated proteins that interacted with IPO11 by mass spectrometry. We identified 14 proteins, with a BFDR (Bayesian False Discovery Rate) <0.02, that preferentially interacted with IPO11 over the Flag-BirA* control (Fig. 5a). Four of the interactors (POM121C, POM121, NUP50, and NUP153) are known members of the NPC, consistent with the role of IPO11 in importing protein cargo through this pore complex. Consistent with a previous high-throughput analysis, two highly similar paralogues: basic leucine zipper and W2 domains 1 and 2 (BZW1 and BZW2, respectively) were also detected as IPO11 interactors [34]. BZW1 and BZW2 are members of the bZIP super family of transcription factors whose function has yet been fully described. BZW1 and BZW2 showed increased expression levels in AML samples as compared with normal hematopoietic cells (Haferlach AML cohort [16]) (Fig. 5b). Similar to IPO11, BZW1 protein was increased in AML compared to normal hematopoietic cells (Fig. 5c) and only detected in the CD34+ fraction of 8227 cells (Fig. 5d). Unfortunately, we were not able to reliably detect BZW2 protein despite using several commercial antibodies (Supplemental Table S4).

Importins facilitate the import of their cargo into the nucleus. Following the BioID finding that IPO11 interacted with BZW1 and BZW2, we tested whether IPO11 knockdown affected the nuclear localization of BZW1 or BZW2. By immunoblotting on nuclear and cytoplasmic fractions and confocal microscopy, the levels of BZW1 were decreased in the nuclei after IPO11 knockdown (Fig. 5e, f). Analysis of the predicted nuclear localization signal for BZW1 and BZW2 is presented in Supplemental table S5.

#### Dual knockdown of BZW1 and BZW2 mimics the effects of IPO11 knockdown

**Fig. 6:**
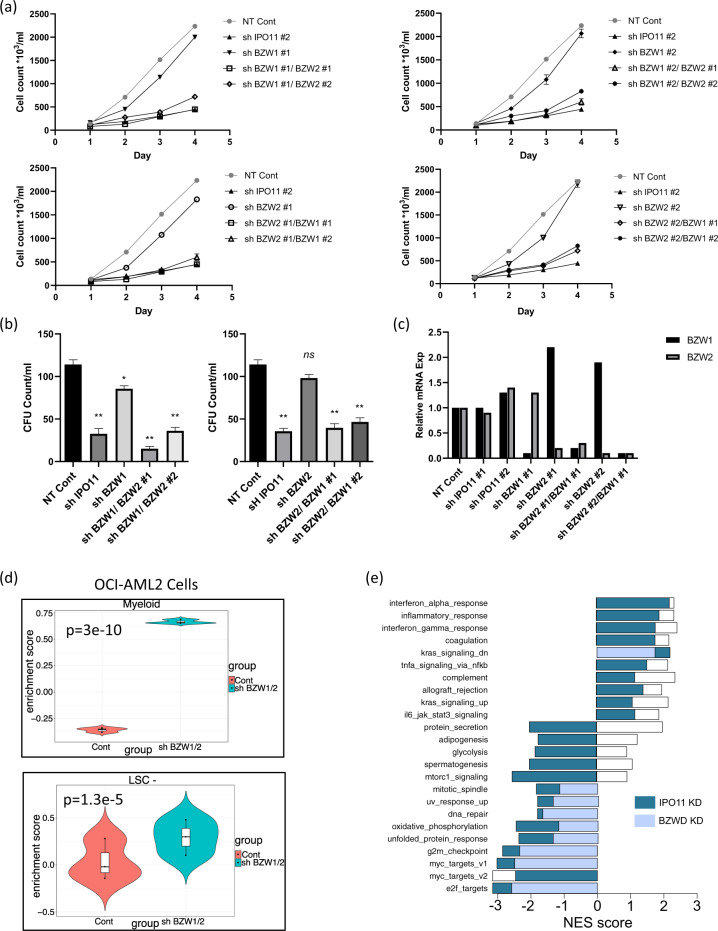
Dual knockdown of BZW1 and BZW2 reduces growth of AML cells and progenitors. **a** OCI-AML2 cells were transduced with shRNA targeting BZW2 or BZW1. Seven days post-transduction cells were transduced with shRNA targeting BZW1 or BZW2, to generate knockdown of BZW1 and/or BZW2. Four days post-transduction with the second shRNA, cell viability and proliferation were measured with an automated fluorescent cell counter. **b** Colony formation from the above BZW1/2 knockdowns. Mean ± SD colony counts are shown per ml (750 cells). *p ≤ 0.05, **p ≤ 0.01. **c** qPCR of the above transduced cells showing levels of BZW1 and BZW2. Please refer also to Fig. S5 for immunoblot analysis of BZW1 in the above samples. **d** Violin plots for myeloid granulocyte (DMA) and LSC- (non-engrafting, myeloid) gene expression after BZW1 and BZW2 dual knockdown in OCI-AML2 cells. **e** Pathway analysis of differentially expressed genes after IPO11 and dual BZW1 and BZW2 knockdown.

Next, we tested whether the nuclear import of BZW1 and BZW2 was functionally important in AML stem cell function and differentiation. Individual knockdown of BZW1 or BZW2 did not alter AML growth and viability or clonogenic growth. However, the dual knockdown of BZW1 and BZW2 reduced AML growth and viability, mimicking the effects of IPO11 knockdown (Fig. 6a). Dual knockdown of BZW1 and BZW2 also reduced the clonogenic growth of AML cells (Fig. 6b, c and Supplemental Fig. S6). We compared changes in gene expression after dual BZW1 and BZW2 knockdown with changes in gene expression after IPO11 knockdown. Dual BZW1 and BZW2 knockdown produced a similar enrichment in genes associated with the LSC- population and granulocyte differentiation as observed after the IPO11 knockdown (Fig. 6d), Likewise, by pathway analysis, knockdown of IPO11 and BZW1/BZW2 showed similar changes, including pathways related to cell cycle regulation, DNA damage response and MYC target genes (Fig. 6e).

#### ChIP-seq analysis of BZW1 and BZW2

**Fig. 7:**
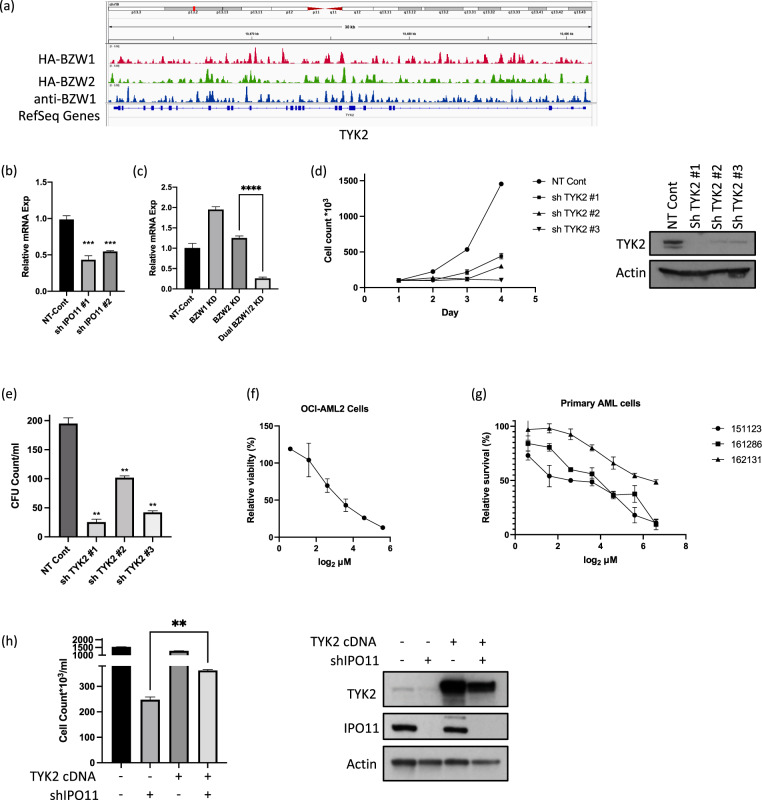
BZW1/2 KD reduces TYK2 levels. **a** ChIP-SEQ analysis using anti-BZW1 antibody and anti-HA antibody in cell transfected with either HA-tagged BZW1 or HA-tagged BZW2. Significant peaks identified TYK2. **b** OCI-AML2 cells transduced with sh against IPO11 and analyzed for TYK2 levels by qPCR. **c** OCI-AML2 cells transduced with sh against BZW1, BZW2, and dual BZW1 and analyzed for TYK2 levels by qPCR. **d** OCI-AML2 cells were transduced with shRNA targeting TYK2 or control sequences. Cell viability and proliferation were measured over time. TYK2 and actin expression were measured by immunoblotting. **e** Clonogenic growth of OCI-AML2, cells transduced with shRNA targeting TYK2 or control sequence. Mean ± SD colony counts are shown per ml (750 cells). *p ≤ 0.05, **p ≤ 0.01, by *t*-test. **f** OCI-AML2 cells were treated with TYK2 inhibitor for 72 h and assessed for growth and viability by XTT-colorimetric based assay. The presented experiment is representative of three biological repeats. Relative survival is calculated in relation to DMSO control in similar concentrations. **g** Primary AML cells derived from three different patients were treated with similar concentration of TYK2 inhibitor and assessed for growth and viability. **h** OCI-AML2 cells were transduced with TYK2 cDNA or empty vector control. Following selection of a stable population, cells were transduced with shRNA targeting IPO11 or control sequences. Equal number of cells were plated and the number of viable cells 5 days after seeding were counted. Levels of TYK2 and IPO11 were measured by immunoblotting.

As BZW1 and BZW2 have a bZIP domain and can act as transcription factors, we performed Chromatin immunoprecipitation sequencing (ChIP-seq) for BZW1 and BZW2. We over-expressed HA-tagged BZW1 and BZW2 in OCI-AML2 cells and ChIP-seq was performed using anti-HA antibodies. We then compared our list of genes enriched in the ChIP for both BZW1 and BZW2 with the genes that were downregulated in the dual BZW1/2 knockdown from the RNAseq, and identified Tyrosine Kinase 2 (TYK2) as a top target. To validate these findings, we also performed ChIP-seq with an anti-BZW1 antibody that immunoprecipitated the endogenous BZW1 and again identified TYK2 as a significant hit (Fig. 7a).

TYK2 is a member of the Janus Kinase family, linked to inflammation and growth factor signaling [35]. We analyzed the expression of TYK2 in OCI-AML2 cells following knockdown of IPO11 or dual BZW1/2 knockdown and demonstrated a significant reduction in TYK2 mRNA levels (Fig. 7b, c). The importance of TYK2 for AML viability, was confirmed as knockdown of TYK2 reduced the growth and viability and clonogenic growth of AML cells (Fig. 7d, e). In addition, the preferential small molecule TYK2 inhibitor, BMS-986165 [36], reduced the growth and viability of OCI-AML2 cells and cells from three different primary AML samples (Fig. 7g). Finally, overexpression of TYK2 partially rescued the loss of AML growth and viability after IPO11 knockdown (Fig. 7h).

## Discussion

By combining a functional CRISPR screen with gene expression of AML stem cells, we identified IPO11 as a novel potential target in AML. Of importance the expression of IPO11 is higher in LSCs compared to bulk leukemia cells. Furthermore, the level of IPO11 was higher at relapse. By overlaying genes that were significant hits in our CRISPR assay, with genes enriched in LSC and upregulated at relapse, we identified IPO11 as the single common hit. Confirming the importance of IPO11, its silencing by knockout or shRNA reduced growth and viability and induced differentiation of AML cells.

IPO11 was initially identified by Plafker et al. as a novel member of the importin family [26], regulating the nuclear localization of protein cargo. Importin β1, the canonical importin protein, was also identified as an essential gene for AML by our CRISPR screen. However, as importin β1 is a central mediator of nuclear protein import with a very wide range of cargo, including transcription factors, cell signaling, and cell cycle proteins among others, attempts to develop inhibitors to importin β1 as novel treatment for cancer have been unsuccessful to date due to their broad toxicity; this is likely due to the wide range of essential proteins they facilitate [37, 38]. In comparison, the relatively narrow range of IPO11 cargo, as demonstrated by our BioID studies and their overexpression in AML cells and stem cells may make IPO11 or its cargo a more favorable therapeutic target.

Through BioID and mass spec we identified BZW1 and BZW2 as cargoes of IPO11. Indeed, dual knockdown of BZW1 and BZW2 mimicked the effects of IPO11 knockdown with reduced growth and viability of AML cells and promoted differentiation. Our results are similar to the findings of previously reported high-throughput screens, which identified BZW1 and BZW2 as potential IPO11 cargo when they surveyed the binding partners of importin family members [39]. Similar to other importins, IPO11 bears an N-terminal Ran-binding domain that specifically binds Ran GTPase, resulting in dissociation of its cargo. Unlike other importins, IPO11 is unique in its ability to recognize Ub-conjugated cargo [27, 40], adding an additional layer of functional regulation to IPO11-mediated nuclear import. Future work will determine how IPO11 binds BZW1 and BZW2 and whether binding is influenced by ubiquitination of these cargo.

BZW1 and BZW2 are highly similar proteins that share a C-terminal W2 HEAT domain and N-terminal zipper domain and may function as transcription factors [41]. Knockdown of either BZW1 or BZW2 had no effect on AML growth or differentiation, suggesting these proteins have redundant functions. To date, little is known about the function of BZW1 and BZW2. BZW1 and BZW2 function in the cytoplasm to regulate protein translation through their C-terminal W2 domains, similar to the eI5 translation factor [42]. By regulating protein translation, BZW1 and BZW2 enhances the translation of ATF4, a key protein in the ER stress response [42, 43], and control ERK/MAPK [44] and Akt/mTOR [45] signaling pathways. Consistent with our data indicating BZW1/2 can act as transcription factors, a prior study demonstrated that BZW1 enhances histone 4 transcription, possibly through its bZIP domain. Our data support a nuclear function for BZW1 and BZW2 in regulating the expression of TYK2. As the overexpression of TYK2 only partially rescued AML cells from IPO11 knockdown, it is likely that there are other important nuclear targets of BZW1/2 and/or IPO11 cargo.

TYK2 is a ubiquitously expressed soluble tyrosine kinase that activates the signal transducer and activator of transcription (STAT) pathway and has been mainly linked to immune or inflammatory responses [46, 47]. Recent studies have highlighted TYK2 can act as an oncogene, with TYK2 activation increasing STAT1 phosphorylation and the expression of BCL-2 in T-cell acute lymphoblastic Leukemia and osteosarcoma [48, 49]. Our data support a role for TYK2 in AML and further exploration of it as a therapeutic target in AML.

In conclusion, we combined a functional genetic screen with gene expression data to identify IPO11 as an essential gene for AML and LSC viability. These data highlight new mechanisms controlling AML stem cell function and differentiation. Moreover, we highlight nuclear protein import as a potential new therapeutic target for the treatment of AML.

## Supplementary information

Supplemental Material

Supplementary Table S1
