# Supplementary material for: IPO11 regulates the nuclear import of BZW1/2 and is necessary for AML cells and stem cells
Source: Leukemia. 2022 Feb 12;36(5):1283–95. doi: 10.1038/s41375-022-01513-4 (PMC9061300; doi:10.1038/s41375-022-01513-4)
Supplement: Supplementary file 1 — Supplemental Material [file 41375_2022_1513_MOESM1_ESM.pdf]

Bioinformatic Analysis

Haferlach AML cohort

Affymetrix gene expression data of AML (542 samples) and healthy bone marrow samples (73 samples) from the Haferlach dataset (GSE13159) was downloaded from the leukemia gene atlas (http://www.leukemia-gene-atlas.de)[16]. The platform used is Affymetrix Human Genome U133 Plus 2.0 Array and the data values correspond to Robust Multichip Average (RMA) expression measure. Official gene symbols from the Hugo Gene Nomenclature Committee were retrieved from the Affymetrix probe identifiers using the R package 'biomaRt'. Data was reduced at the gene level by selecting the probe with the highest median absolute deviation (MAD) across samples per gene. In order to study the pattern expression of IPO11 between AML, normal patients and within the AML samples, data were centered, scaled and clustered using the heatmap.2 function available from the gplots R package. Using the default parameters, each row (gene) in the result has mean 0 and sample standard deviation 1. Hierarchical clustering of the samples was done with the Euclidean distance metric and the complete linkage method on the scaled data. Data distribution of AML, normal samples and the four cluster groups were visualized using boxplots of scaled data.

Diagnosis/Relapse AML cohort.

Gene expression between diagnosis and relapse AML samples were obtained from a previous study by Shlush *et al.*[2]. TMM-normalized counts between diagnosis and relapse were compared using the edgeR likelihood ratio test and tests were corrected for multiple hypothesis testing using the Benjamini-Hochberg procedure. An expression score that

ranks genes from top up regulated in the relapse samples to top down regulated when compared to diagnosis was calculated using the formula `-log10(pvalue) * sign(logFC)` ROc and ROp categories were taken from the above-mentioned work by Shlush *et al.*[2] to categorize these AMLs into progenitor-like and myeloid-like respectively.

TCGA cohort.

Raw counts from 179 AML samples from the LAML TCGA data were downloaded from the GDC portal (https://portal.gdc.cancer.gov/). AMLs were categorized into Roc/progenitor-like or Rop/myeloid-like as described[2]. TMM-normalized counts between Roc/progenitor-like and Rop/myeloid-like categories were compared using the edgeR likelihood ratio test and tests were corrected for multiple hypothesis testing using the Benjamini-Hochberg procedure. An expression score that ranks genes from top up regulated in the Roc/progenitor-like fractions to top down regulated when compared to Rop/myeloid-like was calculated using the formula `-log10(pvalue) * sign(logFC)`.

beatAML cohort

CPM normalized counts of 451 AML samples were obtained from Tyner *et al.*[17].

LSC+/LSC- and CD34+CD34- AML gene expression data

Illumina beadchip transcriptomics data containing LSC+, LSC-, CD34+ and CD34- sorted AML fractions were obtained from the Gene Expression Omnibus data portal (GSE76008)[18] and differential expression between LSC+/LSC- and CD34+/CD34- fractions was calculated using a moderated t test available in the limma R package 3.28.21 incorporating array batch effects in the linear model. An expression score that

ranks genes from top up regulated in the LSC+ fractions to top down regulated when compared to LSC- fractions was calculated using the formula `-log10(pvalue) * sign(logFC).

Overall survival analysis:

IPO11 expression from TCGA and BeatAML datasets were divided into 2 groups – high and low – using a median split. Samples were divided to 3 categories: poor Cytogenetic risk, LSC17 score high or LSC17 score low. The proportions over the total number samples were calculated and the significance of the difference between the proportions between the IPO11 low and high groups was estimated using the Pearson's chi-squared test statistic using the R prop.test function.

Plasmids and transduction

shRNA knockdown

The coding sequence of shRNAs and the control shRNA targeting the GFP sequence (GFP, accession number clonetechGfp_587s1c1) are as follows:

IPO11 (accession no. NM_016338):

shRNA 1 (CDS region):

5'-CCGGAGATCAGTTTCTACCGTATTTCTCGAGAAATACGGTAGAAACTGATCTTTTTG

shRNA 2 (3UTR region):

5'-CCGGGCTGAGATGAAGAAATCACTTCTCGAGAAGTGATTTCTTCATCTCAGCTTTTTG

BZW1 (accession no. NM_ 014670)

shRNA 1 (CDS region):

5'- CCGGGCAGTAGCTAAGTTTCTTGATCTCGAGATCAAGAAACTTAGCTACTGCTTTTTG

shRNA 2 (CDS region):

5’- CCGGGCAGAAACACTCTTTGACATTCTCGAGAATGTCAAAGAGTGTTTCTGCTTTTTG

BZW2 (accession no. NM_014038):

shRNA 1 (CDS region):

5’- CCGGCAGAAGATTGTGGTTCTCTTTCTCGAGAAAGAGAACCACAATCTTCTGTTTTTG

shRNA 2 (CDS region):

5’- CCGGGCTGATGTTCTGAGCGAAGAACTCGAGTTCTTCGCTCAGAACATCAGCTTTTTG

TYK2 (accession no. NM_003331):

shRNA #1(CDS):

5’- CCGGCGAGCACATCATCAAGTACAACTCGAGTTGTACTTGATGATGTGCTCGTTTTTTG

shRNA #2 (UTR):

5’- CCGGGATGTCAGCCTCACCCACACCCTCGAGGGTGTGGGTGAGGCTGACATCTTTTTTG

shRNA #3 (UTR):

5’- CCGGCGTGAGCCTAACCATGATCTTCTCGAGAAGATCATGGTTAGGCTCACGTTTTTTG

Lentiviral packing

The above mentioned vectors (The hairpin-pLKO.1, pLKO, pLentiEF1a, pRS19 and pLENTI-C-HA) were isolated using the E.N.Z.A® Plasmid Midi Kit system (Omega bio-tek), and then quantified by the NanoDrop™ (ThermoFisher Scientific) spectrophotometer. Lentiviruses were made in a 25 cm^2 flask format, by transfecting packaging cells (293T) with a three-plasmid system (gag, pol, and rev genes, and envelope plasmid), as previously described[5]. For 8227 cells and primary AML cells transduction, lentivirus was made in a 175 cm2 flask format, by transfecting 293T cells

with a three-plasmid system. The virus was concentrated using the Lenti-X Concentrator as per manufacturer's instructions.

Transduction of AML Cell Lines

To perform lentiviral transductions, 1.0x10^6 OCI-AML2 cells were centrifuged and resuspended in 3 mL of medium containing 5 µg/mL of protamine sulfate. 1 mL of virus was added to these cells, 5 x 10^6 NB4 and TEX cells were resuspended in 5 mL of medium containing 5 µg/mL of protamine sulfate. 2 mL of virus was added to TEX cells and 1mL to NB4 cells, followed by an overnight incubation (37°C, 5% CO_2). The following day, fresh medium with puromycin (1.5 µg/mL for OCI-AML2 and NB4 or 2 µg/mL for TEX) was added to cells. Three days later, the medium was replaced with non-puromycin containing media. For CAS9-OCI-AML2 cells transduced with IPO11 CRISPR-sgRNA knockout, culture was continued for additional 7 days in a selection-free medium prior to analysis.

For dual BZW1/ BZW2 knockdown, OCI-AML2 cells were initially transduced with shBZW2 pLKO MISSION custom vector with G418 selection (Sigma Aldrich, Missouri, USA). Cells were cultured in G418 (1.5 µg/ml) for 6 days, and then transduced with shBZW1 pLKO-Puro vector, as described above. Dual knockdown was confirmed by qPCR.

Transduction of primary AML cells and 8227 cells

24-well plates were coated with 250 µL of RetroNectin® (20 µg/mL in PBS) per well for 2 hours at room temperature. The wells were blocked with 1 mL of 2% BSA (W/V) for 30

minutes at room temperature. The BSA solution was then aspirated, and concentrated lentiviral particles in HBBS with 25 mM HEPS were added to each well at a volume of 0.5 mL. The plate was then centrifuged at 3,000 rpm for 5 hours at room temperature to promote the attachment of lentiviral particles to RetroNectin. Next, 0.4mL of the viral supernatant was aspirated. 5x 10^5 cells were added to each well in 1 mL of X-VIVO 10 (20% BIT, 50 ng/ml Flt3-L, 10 ng/mL IL6, 50 ng/mL SCF, 25 ng/mL TPO, 10 ng/mL IL3, 10 ng/mL G-CSF). The plate was then centrifuged again at 1,300 rpm for 10 minutes at room temperature to promote the interaction between the cells and lentiviral particles, and then transferred to a 37°C incubator to initiate lentiviral transduction. Twenty-four hours afterward, the cells were resuspended in fresh media at a concentration of 1x 10^6 cells/mL and seeded in a 24-well plate at 1 mL per well. Knockdown was confirmed by qPCR following sorting of GFP positive cells at 48 hours post-infection.

qRT-PCR

Total RNA was isolated using the RNeasy Plus Mini Kit (QIAGEN), and cDNA was prepared using SuperScript IV Reverse Transcriptase (ThermoFisher, MA, USA). Equal amounts of cDNA for each sample were added to a prepared master mix (Power SYBR Green PCR Master mix; Applied Biosystems, CA, USA). Quantitative real time polymerase chain (qRT-PCR) reactions were performed on an ABI Prism 7900 sequence detection system (Applied Biosystems, CA, USA). To normalize for equal amounts of cDNA we assayed the transcript levels of 18srRNA gene. The comparative CT method was calculated as per manufacturer's instructions.

RNA sequencing and analysis

IPO11 and BZW1/2 KD RNA-Sequencing

RNA was isolated from OCI-AML2 cells 7 days post-transduction with shRNA targeting IPO11 or control and was sequenced (Illumina Nextseq2500). Prior to analysis, read adapters and low-quality ends were removed using Trim Galore v. 0.4.0. Reads were aligned against hg19 using Tophat v. 2.0.11. Read counts per gene were obtained through htseq-count v.0.6.1p2 in the mode “intersection_nonempty”.

Gene read counts were normalized using the TMM method available from the edgeR_3.24.3 package. After removing genes whose cpm (counts per million reads) were less than 0.5 to remove noise, edgeR was used to estimate differential expression by applying a generalized linear model between the control samples and the cells isolated after IPO11 or BZW KD. A score that ranks genes from top up regulated in the KD samples to top down regulated when compared to control samples was calculated using the formula `-log10(pvalue) * sign(logFC)`. Raw data was deposited in GEO repository, GSE173288.

Pathway and Network Analysis

Gene set enrichment analysis (GSEA software, http://software.broadinstitute.org/gsea/msigdb/) was used to compare IPO11KD/control and BZW1.2/control cell samples using the 2 corresponding ranked lists of genes and the Baderlab gene-set file (http://baderlab.org/GeneSets) as the pathway database. GSEA was run using default parameters. GSEA results were visualized as a network using

Cytocape 3.7.1 and Enrichmentmap 3.2.1. AutoAnnotate 1.3.2 automatically labelled pathway modules using most frequent words. LSC+ and LSC- gene lists were added as gene-sets to the enrichment map and the significance of overlap with gene-sets was calculated using the EnrichmentMap post analysis integrated hypergeometric test. GSEA was also used using the LSC+/LSC- rank file the CRISPR and Relapse specific genes selected at FDR 0.05 as gene-sets. Importin network was created using Cytoscape 3.7.1 and the STRING app.

Single sample gene set enrichment analysis (ssgsea) was run using the R package GSVA_1.30.0 using normalized counts per million (CPM) on the TCGA, beatAML and Haferlach cohorts. Sgsea was run against AML derived HSC/ progenitor, and myeloid specific gene lists (Supplementary reference[1]). Enrichment scores were row normalized and visualized as a heatmap using the gplots R package. Samples with a positive enrichment score for the HSC/Progenitor gene list and a negative enrichment score for the myeloid gene list were classified as HSC/Progenitor and the other cases were classified as myeloid.

ATAC sequencing and analysis

ATAC samples were preprocessed according to the ENCODE ATAC-seq pipeline. Single-end reads were aligned to the hg38 genome using Bowtie2 (Supplementary reference[2]), with the–local parameter, reads with MAPQ scores < 30 were filtered out with Samtools (Supplementary reference[3]), duplicates were removed using Sambamba (Supplementary reference[4]) and TN5 tagAlign shifted files were created. MACS2 (Supplementary reference[5]) was used to call peaks with the following parameters: -p 0.01–shift 75–extsize

150–nomodel -B–SPMR –keep-dup all–call-summits. Peaks were later filtered at a q-value threshold of 0.0001 for further analyses. Peak counts and sizes for each replicate were calculated using a custom Python script, and Jaccard indices for similarities between called peaks was calculated using BEDTools (Supplementary reference[6]) Differentially accessible regions were calculated using the DiffBind and EdgeR (Supplementary reference[7]) packages in R. Regions with an FDR value <= 0.05 were defined as significantly differentially accessible regions.

Immunocytochemistry

Cells were seeded on 18mm coverslips and were subjected to treatment 48hrs post-seeding. Following treatments, cells were fixed using 4% paraformaldehyde (Bioshop cat: PAR070) for 15min and permeabilized using 0.1% Triton-X 100 (Sigma cat: X100-5ML) for 10min at room temperate. Coverslips were incubated using a blocking solution comprising of 10% FBS, 5% BSA (Bioshop cat: ALB001), 1% fish skin gelatin (Sigma cat: G7765), and 0.25% Triton X-100 (Sigma cat: X100-5ML) in PBS for 2hrs at room temperature. After blocking, coverslips were incubated with primary antibody, anti-γH2AX (1:500) (Millipore cat: 05-636 JBW301, clone JBW301), diluted in blocking solution overnight at 4oC with gentle agitation. Following washes with PBS + 0.05% Tween 20 (PBST), slides were stained with secondary antibodies conjugated to Alexa-Fluor 488 (Invitrogen cat: A11008, A11001) at room temperature for 2hrs at a concentration of 1:500 while protected from light. Following washes with PBST, coverslips were counterstained with DAPI (Invitrogen cat: D1306) and mounted using MOWIOL (Sigma cat: 81381). Slides were allowed to dry overnight before visualization and analysis. All

immunofluorescence images were taken using a Lecia DM4000 B fluorescence microscope using 63X or 100X magnification. Image J software (FIJI) (National Institutes of Health) was used to process and score γH2AX nuclear staining intensity by first using the DAPI signal to create nuclei masks and measuring fluorescence intensity. 10 random fields of view were measured per experiment. Microscope exposure settings were held constant within each experiment.

References

1. van Galen P, Hovestadt V, Wadsworth Ii MH, et al. Single-Cell RNA-Seq Reveals AML Hierarchies Relevant to Disease Progression and Immunity. *Cell*. 2019;176(6):1265-1281.e24.
2. Langmead B, Salzberg SL. Fast gapped-read alignment with Bowtie 2. *Nat. Methods*. 2012;9(4):357–359.
3. Li H, Handsaker B, Wysoker A, et al. The Sequence Alignment/Map format and SAMtools. *Bioinforma. Oxf. Engl.* 2009;25(16):2078–2079.
4. Tarasov A, Vilella AJ, Cuppen E, Nijman IJ, Prins P. Sambamba: fast processing of NGS alignment formats. *Bioinformatics*. 2015;31(12):2032–2034.
5. Zhang Y, Liu T, Meyer CA, et al. Model-based Analysis of ChIP-Seq (MACS). *Genome Biol.* 2008;9(9):R137.
6. Quinlan AR. BEDTools: The Swiss-Army Tool for Genome Feature Analysis. *Curr. Protoc. Bioinforma.* 2014;47:11.12.1-34.
7. Robinson MD, McCarthy DJ, Smyth GK. edgeR: a Bioconductor package for differential expression analysis of digital gene expression data. *Bioinformatics*. 2010;26(1):139–140.

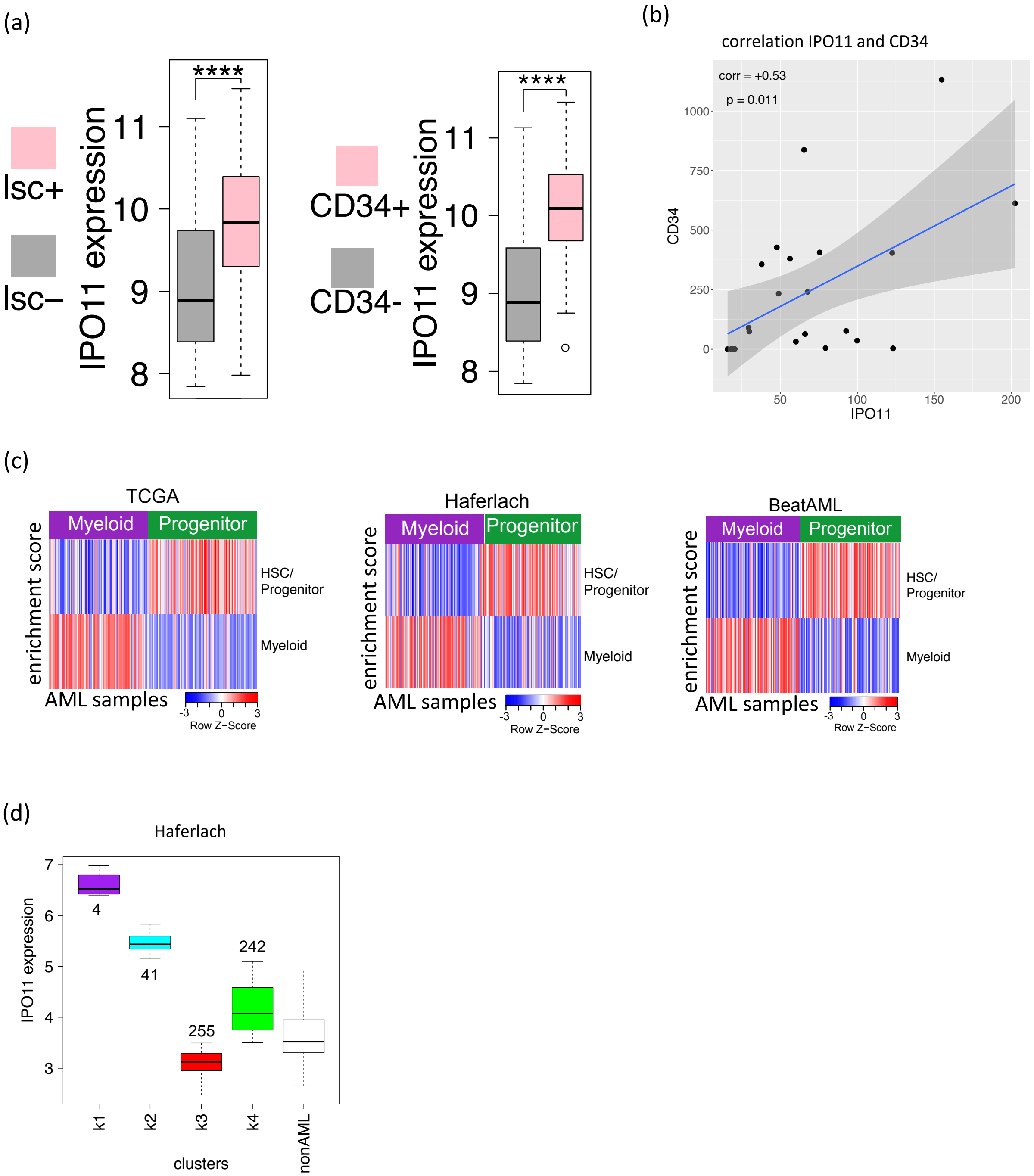

Figure S1: IPO11 expression in AML

(a) Expression of IPO11 expression in LSC+ vs. LSC- and CD34+ vs. CD34- AMLs in the GSE76008 dataset, p value <= 0.0001(****).

(b) Correlation of IPO11 with CD34 gene expression in 11 relapse/diagnosis paired samples.

(c) Heatmap of normalized enrichment score of AML samples classified as myeloid or HSC/progenitor from different cohorts.

(d) Expression pattern and hierarchical clustering of IPO11 gene expression data from 536 primary human AML and 73 normal bone marrow samples (designated non-AML, Haferlach AML cohort). One Way Analysis of Variance and pairwise t-tests were applied to test the significance of the differences in the mean values between the groups, p <0.0001.

Figure S1

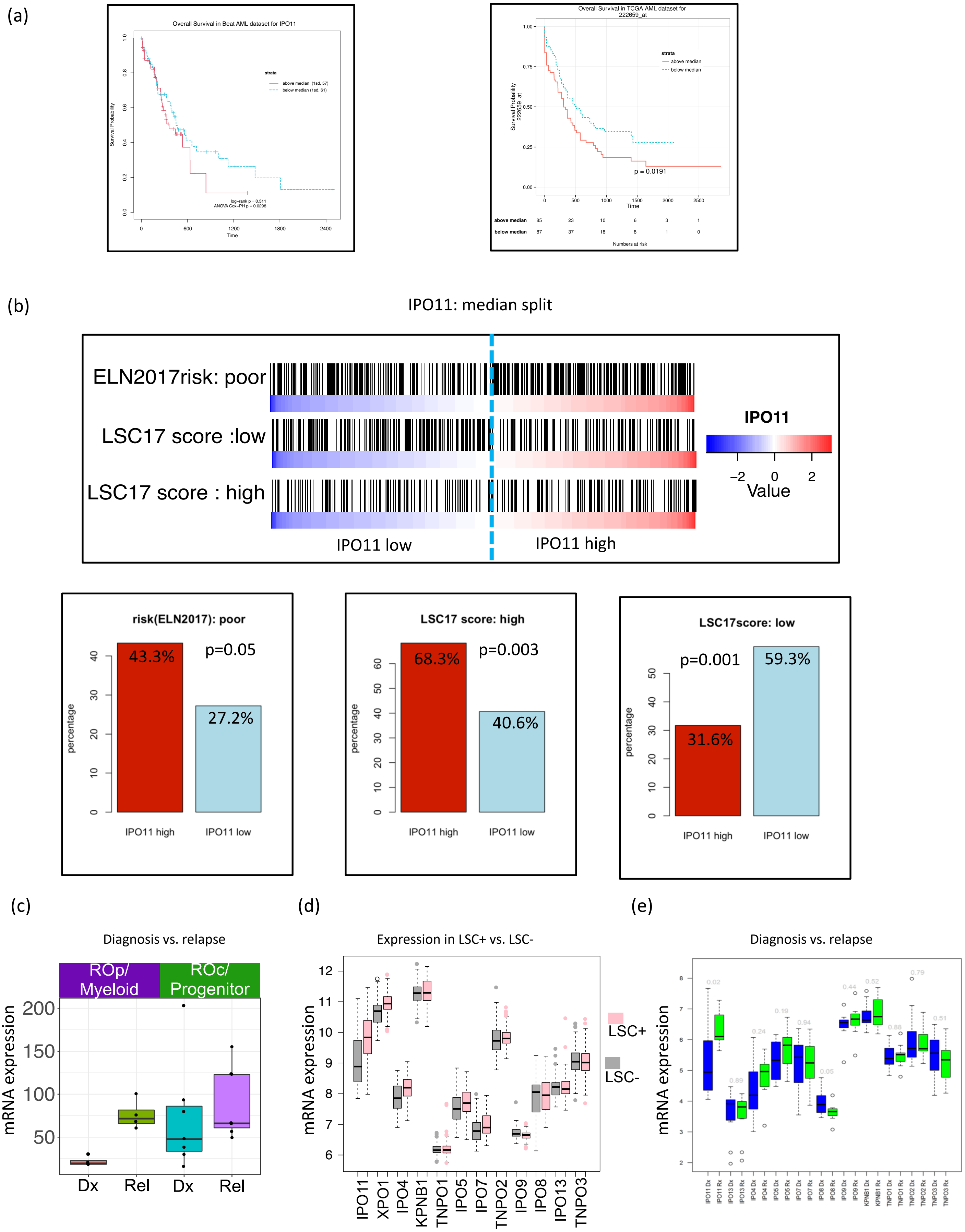

Figure S2: IPO11 expression with regard to overall survival and risk stratification

(a) Overall survival stratified by medianIPO11 expression in the Beat AML and TCGA databases.

(b) IPO11 expression by ELN17 risk stratification and LSC17 score

(c) IPO11 gene expression in 11 paired diagnosis/relapse AML samples fractionated into myeloid-like and HSC/Progenitor like AMLs.

(d) Ranking of importin β family members according to LSC fractions described above.

(e) Analysis of importin β family members expression in paired diagnosis/relapse samples.

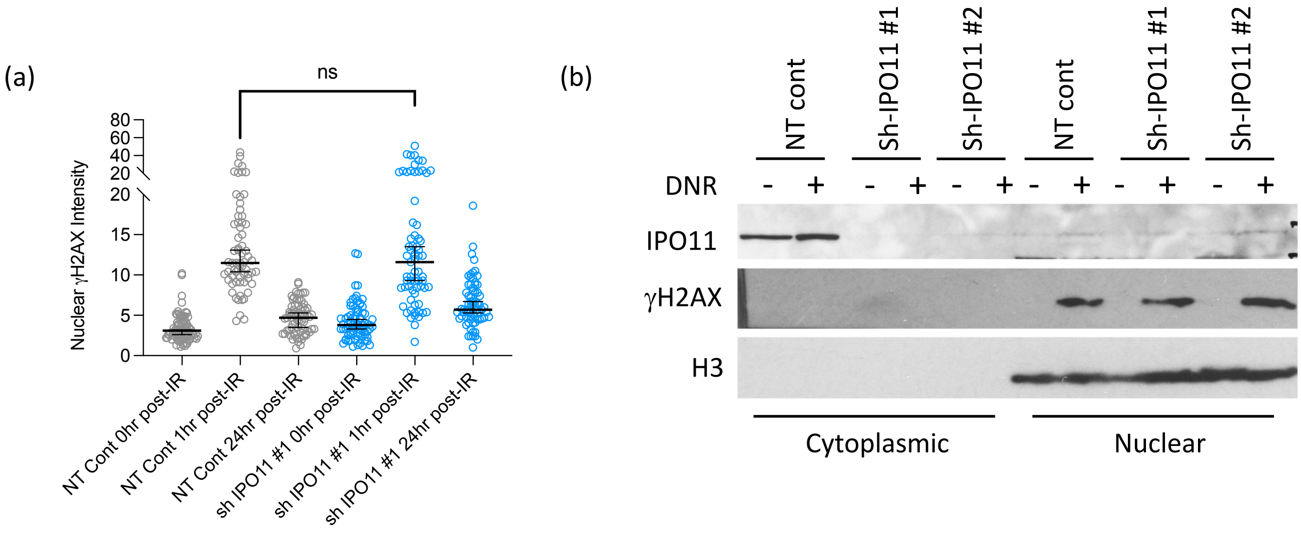

Figure S3: DNA damage response is not affected by IPO11 knockdown

(a) OCI-AML2 cells transduced with shRNA targeting IPO11 or control were irradiated (5Gy) and stained for nuclear g-H2AX. Image J software (FIJI) (National Institutes of Health) was used to process and score γH2AX nuclear staining intensity by first using the DAPI signal to create nuclei masks and measuring fluorescence intensity. 10 random fields of view were measured per experiment.

(b) OCI-AML2 cells transduced with either shRNA targeting IPO11 or control were treated with Daunorubicin 100 nM for 20 hours. Cytoplasmic and nuclear extracts were generated and blotted for IPO11, phosphor- g-H2AX and Histone-3 (H3)

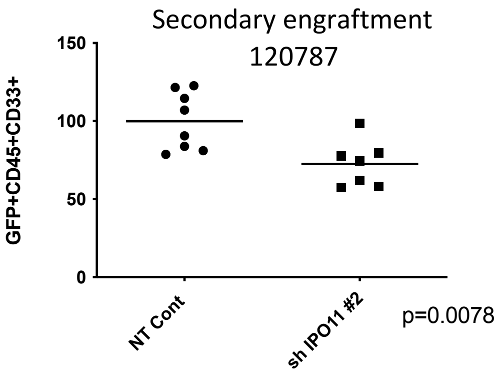

Figure S4: IPO11 is necessary for LSC function

Primary AML cells were transduced with shRNA targeting IPO11 or control sequences in lentiviral vectors containing a GFP marker. 2 days after transduction, equal numbers of viable cells were injected into sub-lethally irradiated NOD-SCID mice preconditioned with anti-CD122. Eight weeks after injection, the percentage of human GFP+, CD33+ and CD45+ cells in the non-injected left femur was determined by flow (see Figure 3e). Same number of cells were further engrafted to assess secondary engraftment.

(a)

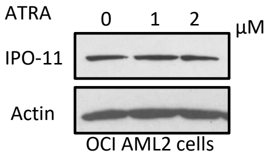

(b)

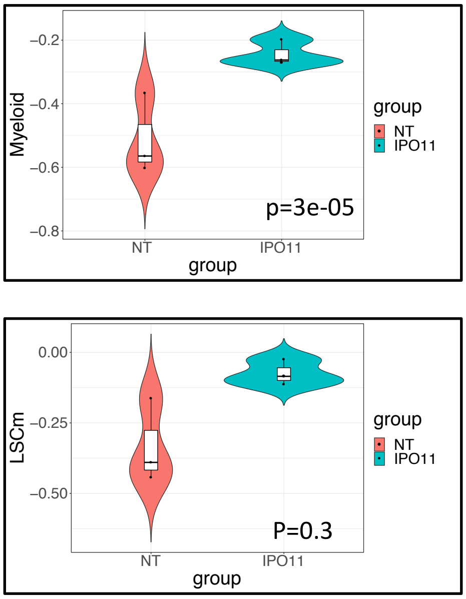

Figure S5: IPO11KD promotes differentiation

(a) IPO11 protein levels 4 hours after treatment of OCI-AML2 cells with increasing concentrations of ATRA.

(b) Violin plots for myeloid granulocyte and LSC- (non-engrafting, myeloid) enrichment scores after IPO11 knockdown in OCI-AML2 cells

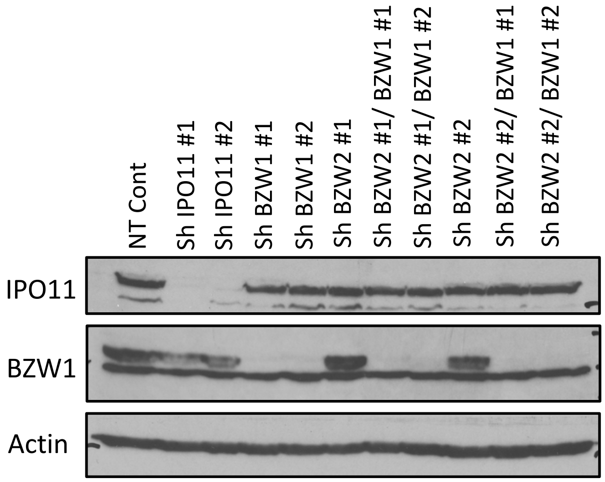

Figure S6: Confirmation of IPO11 and BZW1KD by western blot analysis

Mutated gene	Pearson correlation with gene expressions			Two-sample t-test p-values with mutation		Fisher's exact t-test p-values with mutation		Numbers of mutations	
	Correlation	Correlation test p-values							
		Without plate	With plate	Without plate	With plate	2 groups	3 groups	2 groups (86/84) (down/up)	3 groups (49/77/44) (lower/middle/upper)
**KIT**	0.487	1.73e-11	3.47e-12	9.63e-01	9.86e-01	4.43e-01	1.00e+00	5/2	1/5/1
**DNMT3A**	0.467	1.34e-10	7.48e-13	2.43e-01	9.60e-02	8.60e-01	8.16e-01	22/20	14/17/11
**NRAS**	0.427	6.12e-09	1.12e-05	5.92e-01	5.50e-01	1.00e+0 0	7.18e-01	6/6	5/4/3
**NPM1**	0.425	7.46e-09	6.69e-07	6.72e-05	2.16e-06	1.63e-02	8.99e-04	31/16	24/16/7
**RUNX1**	0.251	9.50e-04	1.38e-02	5.31e-01	8.08e-01	3.04e-01	6.01e-01	6/10	1/13/2
**WT1**	0.227	2.96e-03	3.41e-04	5.39e-01	6.72e-01	1.00e+0 0	2.21e-01	5/5	0/8/2
**TP53**	0.204	7.67e-03	4.00e-02	1.07e-01	1.58e-02	4.01e-01	1.86e-01	5/8	1/8/4
**IDH2**	0.127	9.79e-02	1.26e-03	2.61e-01	7.62e-02	8.03e-01	1.00e+00	8/9	5/8/4
**FLT3**	0.031	6.88e-01	9.21e-01	3.82e-01	1.30e-01	3.97e-01	6.53e-01	27/21	16/20/12
**TET2**	0.03	6.99e-01	1.50e-01	4.69e-01	6.82e-01	4.29e-01	7.04e-01	6/9	3/8/4
**IDH1**	0.013	8.67e-01	4.38e-01	5.98e-01	6.38e-01	4.32e-01	7.44e-01	10/6	6/6/4
**CEBPA**	-0.289	1.29e-04	1.05e-02	6.55e-02	2.83e-03	4.63e-02	1.43e-01	3/10	2/5/6

Table S2: Analysis of molecular mutations in correlation with IPO11 expression in newly diagnosed AML patients

Sample #	Sex	Age at Dx	Diagnosis	FAB Classification	Molecular	Cytogenetics	Sample Status
110661	Female	62	AML	M1	NPM1 positive, FLT3-TKD undetectable, FLT3-ITD undetectable	46,XX [20]	diagnosis
0512	Male	61	AML	M2		46,XY,dup(1)(q21q32)[4]/49,XY,+8,+9,+13[4]/46,XY[12]	diagnosis
100864	Male	42	AML	M6	NPM1 positive, FLT3-TKD positive, FLT3-ITD undetectable	46,XY[20]	relapse
120858	Female	55	AML	M4	NPM1 positive, FLT3-TKD positive, FLT3-ITD positive	46,XX[20]	relapse
120287	Male	77	AML	M1	NPM1 positive, FLT3-TKD undetectable, FLT3-ITD positive	46,XY[20]	diagnosis
140863	Male	70	AML			42~45,XY,-3,-5,del(7)(q21),der(12)t(12;?16)(p13;?11.2),-13,-14,-16,-20,+r,+4mar[cp10]	diagnosis
130414	Male	33	AML	M4	NPM1 positive, FLT3-TKD undetectable, FLT3-ITD positive	46,XY[20]	relapse

Table S3: Patients clinical data

Cat Number	Source	Company
21001-1-AP	Rabbit polyclonal	**Proteintech**
Ab96682	Rabbit polyclonal	**Abcam**
PA5-55386	Rabbit polyclonal	**ThermoFischer**

Table S4: Anti-BZW2 ab tested

The predicted NLS(s) (score cutoff = 0.1)

Protein ID	Predicted NLS	Start	Stop	The highest score of matches within the prediction
BZW1	GQRFKTRKRDEKERFD	13	28	0.685
BZW1	NQQTIGARK	247	255	0.482
BZW1	KDINAVAASLRK	197	208	0.104

The predicted NLS(s) (score cutoff = 0.1)

Protein ID	Predicted NLS	Start	Stop	The highest score of matches within the prediction
BZW2	TGQRFKTRKRDEKEKFEP	10	27	0.681
BZW2	RKANLDKR	208	215	0.435

Table S5: SeqNLS: Nuclear localization signal prediction based on frequent pattern mining and linear motif scoring

**IPO11-F**	**TGCCAGCACTGTTGTTCTTC**
**IPO11-R**	CACGTCTCCAGTAGCGATCA
**BZW1-F**	CAGCAGCTTTTGCTGTGAAG
**BZW1-R**	GGTTTGCTGATTCCGAACAT
**BZW2-F**	CAGGAAGGAACTGCAGAAGG
**BZW2-R**	TGTTCCACTCAACAGCGTTC
**TYK2-F**	CCTCCTGGAGATCTGCTTTG
**TYK2-R**	TCTGGGTTGGCTCATAGGTC

Table S6: Primers used for qPCR analysis
